# The Sanative Influence of Climate; with an Account of the Best Places of Resort for Invalids in England, the South of Europe, &c.

**Published:** 1841-07

**Authors:** 


### Art. XI.

*The Sanative Influence of Climate; with an account of the best places of resort for Invalids in England, the South of Europe, &c.* By Sir James Clark, Bart., m.d. f.r.s., Physician in ordinary to the Queen and to the Prince Albert. Third Edition.—*London,* 1841. *post* 8vo, pp. 377.

For the new edition before us this work, we are informed in the advertisement, has been almost entirely rewritten, and whatever appeared not directly to the purpose omitted, in order to make room for the consideration of several new subjects and the introduction of notices of some places not previously described. The work as it now stands thus presents the quality—rare in a new edition—of being actually less in size and price though even more abounding in information than its predecessors.

The peculiar excellence which at once stamped the character of this work when it first appeared twelve years ago was the philosophical and discriminating manner in which climate was viewed as a remedy. That climate exerts a powerful influence over health and disease is a fact which had been long known and generally recognized; but it must be confessed that its application as a remedy in disease had too often been made without discrimination both as regards the climate selected and the stage at which the disease was sought to be benefited by the change. Change of climate had too often been resorted to as a last resource, or it had been misapplied in cases wherein it would otherwise have been capable of yielding essential service. Patients who really might have derived much benefit from change of climate have too often been sent abroad without proper directions regarding the situation most suited to their complaints, and altogether uninstructed respecting various circumstances, a due attention to which is necessary to give full effect to the best selected climate.

In illustration of the indiscriminate manner in which the choice of a climate used to be made we may instance Montpelier. This place is so associated in the minds of the public of this country with everything that is salutiferous in climate, that the very name is frequently employed as an epithet to any place considered more than usually healthy. In accordance with this notion, Montpelier used to be the place to which consumptive invalids wishing to try the effects of change of climate were almost as a matter of course first directed. The celebrity of the Medical School of Montpelier, says Sir James Clark, had probably a considerable share in giving rise to the character which this place obtained for the benignity of its climate,—*olim Cous nunc Monspeliensis.* But whatever may have been the merits of its medical school, it will be easy to show that the climate little deserved the reputation which it long enjoyed as a residence for the consumptive.

Consumption is the disease in which it has been generally supposed that the beneficial effects of climate are chiefly evinced; but far from this being a correct view of the matter, our author remarks that were the character of climate as a remedy to be estimated by its effects on consumption, it would be justly valued at a very low rate. In dyspepsia, and disorders of the digestive organs generally, and in the nervous affections and distressing mental feelings which so often accompany these; in asthma, in bronchial diseases, in scrofula, and in rheumatism, the beneficial effects of climate are far more strongly evinced than they are in consumption. In cases also of general delicacy in childhood and youth, in the climacteric disease, in convalescence, and in disordered health from various causes, as from a residence in hot climates, change of climate is a valuable remedial agent.

The statistical reports on the sickness, mortality, and invaliding among the British troops in almost all quarters of the globe show that there is no immunity from consumption in any climate, and on this objections to the value of climate as a remedy have been grounded; but Sir James Clark justly observes that when an invalid is sent abroad for his health, he goes, by the direction of his physician, to the climate best suited to his particular case, and at the most favorable season of the year. Moreover, he goes prepared to avail himself of all the advantages of his new situation, and to avoid as far as possible its disadvantages. Hence the influence of any climate upon such an invalid must be estimated very differently from the influence of the same climate on the permanent inhabitants, or on our troops who are resident in it at all seasons, and are exposed to all its prejudicial influences for years. "The great lesson," continues our author, "which the army medical reports teach in regard to consumption is this, that as it is a prevalent and fatal disease in all climates and among all nations, our attention should be chiefly directed, not to a state of disease which is incurable by climate or any other means, but to the prevention and cure of the disordered state of health which constitutes the real cause of consumption. When change of climate is judiciously employed as a remedy for this constitutional disorder which precedes consumption, there will no longer remain any doubts of its beneficial influence, and what a single change of climate does not effect, a succession of changes will often be found to accomplish."

As to the mode of operation of change of climate, our author takes particular pains to show that it is by no means possessed of any specific quality by virtue of which it directly cures the disease. "Let it not be imagined," he says, "that change of climate, however powerful as a remedy, can be considered as at all peculiar in its mode of action, or as justifying on the part either of the physician or patient the neglect of those precautions which are requisite to ensure the proper action of other remedies." Among other collateral advantages attending a residence in a mild climate may be mentioned the very important one, that the patient is permitted to enjoy frequent exercise in the open air, an advantage for which there is no substitute.

In the present edition, the order of the two parts into which the work is divided has been reversed, so that the account of the principal diseases benefited by a mild climate now stands first, and after it comes the consideration of the general physical characters and sanative influence of the climates of the various places which have been recommended for the resort of invalids.

The disorders of the digestive organs are classed under three heads, viz. inflammatory or gastritic dyspepsia; irritable or nervous dyspepsia; and atonic dyspepsia, or that form of the complaint which depends chiefly on a loss of tone. This arrangement is made in reference to the kind of climate which the different forms of the disease require. The patient with gastritic dyspepsia should not, for example, go to Nice nor the south-east of France. In cases of this kind, the south-west of France or Devonshire is preferable, and Rome and Pisa are the best places in Italy. On the other hand, in atonic dyspepsia, in which languor and sluggishness of the system as well as of the digestive organs prevail with lowness of spirits and hypochondriasis, Nice is to be preferred to all the other places mentioned; and Naples will generally agree better than Rome or Pisa; while the south-west of France and Devonshire, and all similar climates, would be injurious. In the nervous form of dyspepsia a climate of a medium character is the best, and the choice should be regulated according as there is a disposition to the gastritic or the atonic form.

The selection of a residence even in the same place, our author further says, is not a matter of indifference to very sensitive invalids. One will feel himself better in an elevated situation, another in a lower and more sheltered one. But dyspeptic patients, who pass the winter in Italy, need not in general be limited to one place. Although the climate most suited to the particular character of their complaint should be selected as their head-quarters, they may visit, during the season, the principal cities in the south of Italy; and if this is done with judgment, the successive changes may prove beneficial to their health. Generally speaking, Rome will be the best residence in Italy in gastritic dyspepsia, especially during the spring; Nice the best climate in the purer cases of atonic dyspepsia.

*Pulmonary consumption.* Under this head, Sir James Clark dwells particularly on the constitutional disorder which precedes, and which he considers the essential predisposing cause of the formation of tubercles in the lungs. This disorder, our readers are aware, he calls TUBERCULOUS CACHEXY, and he considers it in general the only curable stage of consumption. "The attention," he says, "is still too exclusively directed to the pulmonary disease, and too little notice taken of the constitutional affection; although it is only on the proper treatment of the latter that we can rest our hopes of success, all efforts to cure the former having been as yet comparatively of little avail."

*Choice of climate for tuberculous cachexy.* With regard to the climates of the south of France and of Italy, Sir James Clark observes, that for consumptive invalids in whom there exists much sensibility to harsh and keen winds, and more especially, if immediate vicinity to the sea coast is known to disagree, Rome or Pisa is the best situation for a winter residence. When, on the contrary, the patient labours under a languid, feeble circulation, with a relaxed habit, and a disposition to congestion or hemorrhage rather than to inflammation, and more especially when the sea-air is known by experience to agree, Nice deserves the preference. In cases complicated with gastritic dyspepsia, however, Nice is an improper residence, its climate being decidedly inimical to such a state. The climate of Hyères may be considered as nearly similar to that of Nice. So great is the influence of such a morbid condition of stomach in modifying all other diseases, that Sir James Clark considers it alone sufficient to claim for itself the chief consideration in deciding upon the particular climate.

The climate which of all others the author considers best suited to consumptive patients generally is that of Madeira. There the patient may reside during the whole year, and thus avoid the inconveniences and even risks attending a long journey, to which consumptive patients who pass the winter in Italy are necessarily exposed when leaving it on the approach of summer. The summer climate of the whole shores and islands of the Mediterranean is unsuited to consumptive invalids.

The winter climates in England most favorable to consumptive patients are those of Torquay, Undercliff, Penzance, Clifton, and Hastings. The choice among these places will depend upon the nature of the case, and especially upon the condition of the digestive organs. For persons of an inflammatory constitution, with a disposition to gastritic dyspepsia, Torquay will form the best residence, while it will as decidedly disagree with persons of a very relaxed habit, and subject to copious secretions from the mucous membranes or to atonic dyspepsia. Such patients will bear the climate of Torquay for a very short time only. What has been said of Torquay applies with equal force to Penzance and the other parts of the Land's End. Undercliff, Hastings, and Clifton will form preferable residences in the constitutions referred to.

With respect to the length of time requisite for a consumptive invalid to pass in a mild climate, in order to overcome the disposition to the disease, our author observes that no general rule can be given. When the measure is had recourse to for the removal of the disordered health which precedes tuberculous cachexy, a single winter will be of great benefit and possibly all that may be necessary. When tuberculous cachexy is established, and still more when there is reason to suspect the presence of tubercles in the lungs, several years may be requisite, and in some cases it may be necessary to reside permanently in a mild climate.

In place of sending consumptive patients to pass the winter in a foreign climate, it has been proposed to keep them at home in rooms maintained at a regulated temperature. "With the advocates of such a measure," our author justly remarks, "the state of the lungs appears to be the only consideration; but without improving the general health by exercise in the open air, all our measures directed to the local disease will be of little avail; the removal of the constitutional disorder can alone afford the patient a hope of recovery." Where circumstances, however, preclude the possibility of changing the climate, and the patient is quite unfit to bear exposure to the external air in this country, confinement to apartments properly and equally heated is the best measure which can be adopted. The invalid who has passed some months in an artificial climate established within doors, should, when he ventures out for the first time, do so with proper precautions. The respirator will prove a valuable protection to him in the first instance and for some time afterwards, on any sudden or considerable fall of temperature.

*Bronchial affections.* In no class of complaints is the beneficial action of change of air and climate more speedily manifested than in irritation of the organs of respiration. In the slighter bronchial affections, a change to a very short distance only has often a remarkable effect; coughs ceasing in the course of a few days, which had resisted medical treatment for many weeks. But in protracted cases the disease assumes a more fixed character, and requires a thorough change of climate to produce much effect on it. When the patient is at the same time suffering from dyspepsia, a very frequent complication in bronchial diseases, this should be remedied, as far as possible, before he leaves his own country, otherwise the change, so far from proving beneficial, may be injurious to him. The skin will also require particular attention, as it is seldom in a healthy condition in persons who have long laboured under bronchial irritation.

With respect to the best winter residence, Sir James Clark found Rome agree more decidedly with such patients than any other place on the continent; and he repeatedly had occasion to compare its influence with that of other climates upon the same patients. The climate of Rome is not, however, so beneficial, when the disease is accompanied with copious expectoration and a relaxed state of the system, as that of Nice; but in the dry tracheal and bronchial affections, the climate of Rome, and also that of Pisa, is preferable. Rome has several obvious advantages over the other residences on the continent for patients labouring under bronchial irritation. It is little liable to high winds, the air is soft, and the surrounding country well adapted for riding—the best exercise for such patients. With the exception of cases in which there is a copious expectoration and a relaxed state of the system, the climate of Madeira proves very beneficial and is preferable, our author thinks, to any part of the continent. In this country Torquay is the best climate in the dry bronchial irritations; for those with copious expectoration, a relaxed condition of the system, or an atonic state of the digestive organs, Undercliff and Clifton afford better climates.

*Asthma.* In no disease, perhaps, is the effect of change of climate so conspicuous as in asthma. Taking the disease generally, it may be stated that a removal to a warmer climate is highly beneficial; but the degree of relief will depend greatly upon the climate being suited to the particular case. We must not, therefore, says our author, prescribe for a name, but take into account the whole pathological condition of the patient, in order that we may be enabled to fix upon the climate best suited to his case. The following forms of asthma our author points out as requiring attention in prescribing change of air or climate: pure nervous asthma; humid asthma; cardiac asthma.

*Rheumatism.* A residence for some time in a mild climate proves of the greatest benefit in chronic rheumatism. Nice and Rome are the best climates on the continent, according to our author's experience.

*Delicacy of constitution, &c.* In cases of delicacy in childhood, a residence for some time in the south of Europe has appeared to our author particularly useful. Rome and Nice he found the best winter residences for children: the former when the digestive organs were in an irritable state, the latter when there was a torpid, languid state of the constitution. About the age of puberty it frequently happens that the health declines, and sometimes tuberculous disease shows itself for the first time. In such cases a temporary residence in a mild climate is of great service. As age advances also, and the system begins to feel the weight of years, a milder climate proves highly beneficial in arresting premature decay. Persons just returned to Europe, and whose constitutions have suffered by a long residence in a tropical climate, will find great advantage in spending one or more winters in the south of Europe, before finally settling in this country. There are various other states of impaired health in which change of air and climate prove very beneficial. Indeed it would be difficult, says our author, to name the chronic complaint or the disordered state of health which would not admit of being ameliorated by the judicious adoption of such a measure. In convalescence from fevers and other acute diseases, no remedy is so effectual in promoting the recovery of the invalid as a well-timed change of air. In short, the author con tinues, change of air is to the young and delicate the best and often the only admissible tonic.

To the observations on the dietetical and therapeutical measures necessary to aid the operation of climate we would particularly call attention. They are sketches merely, but they bear the mark of the master's hand. Our limited space prevents us from doing more than noticing those on the treatment of dyspepsia in children. This is a disease which so frequently undermines the future health, that it is of the utmost importance that correct views should be entertained respecting it. Change of climate, though when properly applied of the utmost advantage to the child, should not be adopted till the morbid state of the digestive organs is in some degree corrected; and wherever a child goes, this, says our author, should receive constant attention. For although the general health may be much improved by change of air, or climate, the improvement will not be permanent unless the congestion and irritation of the digestive organs, in which the disorder had its origin, and on which its continuance depends, are removed. The most important means of correcting this state is the regulation of the diet, which of course must be varied according to the age of the child and the degree of congestion and irritation which exists. Generally speaking, the diet should be of the blandest quality, more especially in children of an excitable constitution. When the tongue is red, the skin hot at night, with thirst, milk and farinaceous food should constitute almost the sole nourishment. As the irritation abates, a little mild animal food every second day is allowable. For children of a more torpid character of constitution, who have little disposition to fever, when the tongue is loaded and all the functions languid, a more exciting diet may be permitted. The warm bath and friction will be beneficial in all cases, more especially in the languid constitutions just alluded to. The great objects in the treatment should be, to regulate the diet according to the sensibility and power of the digestive organs, to promote an active state of the circulation on the surface and extremities, with a view to remove the congestion and irritation of the internal organs, and to impart tone to the system. This irritation of the digestive organs influences every function of the body, and without its removal, all remedies directed to the improvement of the general health will produce only a partial and evanescent effect.

In alluding to the medical treatment of such cases, Sir James Clark animadverts on the inconsiderate routine practice generally followed. Active mercurial purgatives, he says, an exciting diet of animal food, not unfrequently repeated several times a day, with the addition of porter or wine, or both, and this followed by steel and other tonics, constitute generally, in this country, the treatment of scrofulous children. Such a mode of treatment, he continues, is at total variance with the gastroduodenal irritation and hepatic congestion, which are present in a greater or less degree in all cases of scrofula. Besides this, a complete want of success attends it in practice, whilst striking benefit is derived from an opposite plan of treatment.

Part Second, *on Climates,* is appropriately introduced with observations on ventilation and unhealthy residences, and directions for invalids making a change of climate. The climates of England, of France, of Nice, of Italy, of Malta, of the Atlantic, are successively treated of; and in an appendix the climates of the Southern Hemisphere.

The mild region of England admits of being divided into four districts or groups of climate; that of the south coast, comprehending the tract of coast between Hastings and Portland Island; the south-west coast, from the latter point to Cornwall; the district of the Land's End; the western group, comprehending the places along the borders of the Bristol channel, and estuary of the Severn. Each of these regions has some peculiar characteristic features, both as regards physical and medical qualities.

*London.* A notice of the climate of London is first given as a point of comparison, and for other reasons. There are some peculiarities in the climate of London owing chiefly to artificial circumstances. The mean annual temperature of London is about 1½° above that of the environs, but this difference of temperature is very unequally distributed throughout the year and throughout the day. The excess of the city temperature is greater in winter than in summer, and belongs in *strictness* to the nights. The heat of the day in London indeed according to Howard, falls, on a mean of years, about a third of a degree short of that in the open plain. The temperature of London, also, has not such an extensive range as that of the environs, and it acquires and loses its heat more slowly. The benefit so often experienced by delicate invalids on coming from the country to London in the winter and spring, is no doubt, our author remarks, owing to the qualities of the climate above enumerated. It is during the night that the climate possesses the greatest advantages for the sensitive invalid; in addition to its warmth and dryness the atmosphere is then in its purest state.

*South coast.* Were we to rest contented with the result of the mean annual temperature, we should find very little difference between that of the south coast and of London. But when we descend to particulars, we observe that there does exist a considerable difference in their temperature, arising from the mode of its distribution. It is because the higher degree of the temperature of London and the interior of the island in summer compensates for the lower degree in the winter, that the climate of these places appears to equal that of the south coast. The mean temperature of the latter, however, during the winter months, is from one to two degrees above that of London. In steadiness of climate, as deduced from the variation of temperature between successive days, the south coast does not appear to possess any very remarkable superiority over London.

More rain falls on the south coast than at London, the ratio being, as nearly as could be ascertained, as 30 to 25; but the quantity varies considerably at different parts. Of the places on this coast frequented by invalids, Hastings, Brighton, and Undercliff may be considered as having respectively peculiar climates. That of Hastings is mild and soft, whilst that of Brighton is dry, elastic, and bracing. Undercliff has somewhat of an intermediate character: it is evidently one of the warmest climates in Great Britain, and most eligible for a large class of invalids.

*South-western coast.* The south coast of Devon has a winter temperature nearly two degrees higher than that of the coast of Sussex and Hampshire, and from three to four higher than that of London. The principal places adapted for invalids on the south-west coast are, proceeding from west to east, Salcombe, Torquay, Dawlish, Exmouth, Salterton, and Sidmouth. The general character of the climate of the south-west coast is mild, soft, and humid. Torquay is that which possesses the advantages of the south-western climate in the highest degree. Torquay is drier than the other places mentioned, and almost entirely free from fogs.

The influence of the south-western climate on disease may be anticipated, our author observes, in a great degree from its physical characters, which are mild but rather humid, consequently soothing, but rather relaxing. In one class of complaints, it is therefore calculated to prove decidedly beneficial; in another, of an opposite character, equally injurious. Pulmonary diseases are those in which the climate has been considered especially beneficial. But for the reasons already pointed out, our author remarks, the benefit to be derived from this climate will depend upon its being applied to the proper cases. In chronic inflammatory affections of the throat, trachea, and bronchi, attended with a dry cough, or with little expectoration, decided benefit may be expected. But when there exists in such cases a relaxed state of the mucous surface, with copious expectoration, especially when occurring in a languid relaxed constitution, the disease is more likely to be aggravated than diminished by a residence on the south-west coast. From this statement will be understood the character of the more serious diseases of the chest, which are likely to be relieved by this climate.

In gastritic dyspepsia it is serviceable; likewise in dysmenorrhœa and the various nervous symptoms consequent upon it. On the other hand, this climate certainly exerts an unfavorable influence on atonic dyspepsia, in all nervous complaints arising from relaxation or want of tone of the nervous system; and on persons subject to menorrhagia and leucorrhœa; and in all diseases of the mucous membranes attended with relaxation and discharge.

What may be the real estimation in which the climate of Devonshire ought to be held in consumptive complaints, and what may be its absolute effect upon these, Sir James Clark has much difficulty in saying: but this much he ventures to advance, that as the invalid will be exposed to less rigorous cold and for a shorter season, will have more hours of fine weather, and consequently more exercise in the open air, he gives himself a better chance by passing the winter here than in the more northern parts of the island. To compare it also, in this respect, with the mild climates of the south of Europe is no easy task. In the south, the invalid has finer days, a drier air, and more constant weather; but the transitions of temperature, though less frequent, are more considerable.

In the night, invalids are often exposed to severer cold in the south of Europe than here.

The climate of the south coast of Cornwall in its general characters, as also in its influence on disease, resembles closely that of the south coast of Devon, and has also long been resorted to by pulmonary invalids. The temperature of Penzance is remarkably equable both throughout the year and throughout the day. The daily range of temperature at Penzance is little more than half that of the south of Europe, though it is more than that of Madeira. Though the mean temperature of the whole twenty-four hours at Penzance is considerably lower than that of the south of Europe, yet during the night through the winter its extreme minimum temperature is seldom so low. Thus the whole advantage of Penzance, it is remarked, as compared with the south of Europe appears to occur in the winter and during the night. The disadvantages which attach to the climate of the Land's End generally, in point of humidity and liability to violent and frequent gales of wind, are such as, in a great measure, to neutralize the superiority which it possesses over the other climates of England, in mildness and equability of temperature.

*West of England.* The vicinity of Bristol and Clifton appears to be the mildest and driest climate in the west of England, and consequently the best winter residence in that part of the country for invalids. Compared with the south and south-west coasts, the spring is the period of the year during which this climate appears to the greatest advantage. This season is warmer here than on the south coast, with the exception of Undercliff, whilst it is equal to the warmer parts of the south-west coast. When the climate of Clifton is compared more closely with that of Devonshire, it may be characterized as drier and more bracing than the latter, and as more exciting to most consumptive patients, and to those labouring under irritable affections of the bronchial membrane. For such cases the softer and more humid air of Devon will be found more soothing while for invalids whose constitutions have suffered from long-continued derangement of the digestive organs, or a congested state of the mucous membranes with copious secretion, and also for young scrofulous persons, and those of relaxed habits of body generally, Clifton will prove a preferable climate. The advantages of Clifton as a residence for the invalid are not limited to the winter: it and its neighbourhood afford also a very favorable summer climate.

*Island of Bute.* Of all places in Scotland the island of Bute possesses the best climate for invalids. The temperature there never falls low during winter, nor rises high in summer; so that its yearly range is comparatively limited. The climate of Bute may be characterized as mild and equable but rather humid. It resembles in character that of the south coast of England and France, and of the Channel Islands, though considerably less warm than any of them. As a winter residence for invalids, it holds out considerable advantages to the inhabitants of Scotland, but to that class of invalids only for whom a soft equable but rather humid atmosphere is indicated.

*Cove of Cork.* This appears to possess one of the mildest climates in Britain; being inferior in point of temperature to Penzance only during the winter months, and to the same place and Torquay only during the spring. In point of dryness, Cove is rather low in the table of comparison. In its general characters of climate and the influence of this on disease, Cove corresponds with the south-west of England and other similar climates.

*The Channel Islands.* The climate of these islands has a close resemblance to that of the south-west coast of England, and especially to Penzance. There are the same equable temperature, the same soft humid atmosphere, and the same liability to high winds during the winter and cold north-east winds in the spring, which characterize the latter place. So close is the affinity of these climates, and so similar their influence on disease, that the remarks which have been made on the south-west of Devonshire and the Land's End, as residences for invalids, are perfectly applicable to the Channel Islands.

*South-west of France.* The southern provinces of France, as regards climate, admit of being classed under two divisions, namely, the south-eastern and the south-western. The climate of the latter resembles in its general qualities that of the south-west of England, being soft, relaxing, and rather humid. Generally speaking, the climate of the south-west of France will be found useful in chronic inflammatory affections of the mucous membranes accompanied with little secretion, as in chronic bronchitis not attended by much expectoration, or difficulty of breathing, and in similar morbid states of the larynx and trachea. It will be equally proper in dry scaly eruptions of the skin; in dysmenorrhœa; in certain kinds of headach, especially those induced or exasperated by sharp north-east winds; and in high morbid sensibility in general, when accompanied with that habit of body which the ancients called *strictum.* On the other hand, the same diseases occurring in relaxed habits, in which there is a disposition to copious secretion, will be aggravated by this climate.

*Pau.* The principal place in this district is Pau. There are several circumstances in the climate of Pau which render it a favorable residence for a certain class of invalids. The atmosphere is generally dry and the weather fine, and there are neither fogs nor piercing winds; the characteristic quality of the climate, however, is the comparative mildness of its spring, and exemption from cold winds—circumstances which render this place favorable in chronic affections of the larynx, trachea, and bronchi. In gastritic dyspepsia, Dr. Playfair has found it beneficial, and he has seen it useful in a few cases of asthma. With delicate children also, he found the climate agree well, especially when they removed to the mountains during the summer. Upon the whole, Pau appears to be the most desirable winter residence in the south-west of France for invalids labouring under chronic affections of the mucous membranes.

Invalids labouring under or subject to attacks of rheumatism should avoid Pau. In bronchial diseases also, when accompanied with much general relaxation of the system, and with copious expectoration and with dyspnœa, the climate will not in general prove beneficial; and Dr. Playfair considers it too changeable in consumptive diseases.

*South-east of France.* Various places in the south-east of France have been at different times recommended as affording a good winter climate for consumptive patients; but nothing can be more unaccountable, Sir

James Clark observes, than how such advice ever came to be given, as the experience of later years is in complete opposition to it, and the general and leading characters of the climate show that there never was the least reason to sanction it. The general character of this climate is dry, hot, and irritating. Its temperature throughout the year and the day is distributed with great irregularity, and the range is much wider than in our own climate. The temperature, however, remains more steady from day to day than our own; but its changes, though less frequent, are more sudden and extensive. Although decidedly improper for consumptive patients, and for those labouring under irritation of the mucous membranes of the stomach, larynx, or trachea, this climate, our author says, may prove useful to invalids of a different class. On persons of a torpid or relaxed habit of body, and of a gloomy, desponding cast of mind, with whom a moist relaxing atmosphere disagrees, the keen, bracing, dry air of Provence and its brilliant skies will often produce a beneficial effect. In some cases of chronic intermittent fevers, also, it proves very favorable. In the following places these distinctive characters of climate prevail more or less, but none can be considered as exempt from them, viz. Montpelier, Marseilles, Hyères. The latter of these places is the least exceptionable residence in Provence for the pulmonary invalid.

*Nice.* In the physical qualities of its climate Nice possesses some advantages over the neighbouring countries of Provence and Italy, inasmuch as it may be said to be free from the sirocco of the latter and protected from the mistral or cold northerly wind of the former.

In consumption, the disease with which the climate of Nice has been chiefly associated in the minds of medical men in this country, little benefit, our author fears, is to be expected from it. But in chronic bronchitis, with copious expectoration, which often simulates phthisis, very salutary effects are produced by a residence in this place. In gout and chronic rheumatism the climate is favorable. Its advantages are also great in scrofulous complaints. On children, the climate generally exerts a very favorable influence if attention be paid to their diet. In the large class of hypochondriacal and nervous symptoms which often originate in dyspeptic complaints, Nice is beneficial. The cases of dyspepsia most benefited are those accompanied with a torpid relaxed state of the system, with little epigastric tenderness, or any of those symptoms which denote an inflamed or very irritable state of the mucous membrane of the stomach: where the latter state prevails, Nice will decidedly disagree. In all cases where there is great relaxation and torpor of the constitution, the climate of Nice is extremely useful. In stating its general influence on the animal economy, Sir James Clark says that the climate of Nice is warm, exhilarating, and exciting, but upon the whole irritating, more especially during the spring, at least to highly sensitive constitutions.

Of the climates of Italy, those of Genoa and Florence have not much to recommend them.

*Pisa.* This place has long had the reputation of possessing one of the most favorable climates in Italy for consumptive patients. Its climate is genial, but rather oppressive and damp. It is softer than that of Nice, but not so warm; less soft but less oppressive than that of Rome. For invalids who are almost confined to the house, or whose power of taking exercise is much limited, Pisa offers advantages over both Rome and Nice.

*Rome.* The climate of Rome is mild and soft, but rather relaxing and oppressive. In regard to its physical qualities, Sir James Clark considers the climate of Rome one of the best in Italy. One peculiarity of it deserving notice is the stillness of its atmosphere; high winds being comparatively of rare occurrence. This quality of calmness, our author remarks, is valuable in a winter climate for pulmonary diseases, and to invalids generally, as it admits of their taking exercise in the open air at a much lower temperature than they could otherwise do. To patients labouring under bronchial irritation, wind is peculiarly hurtful.

Among the diseases benefited by a residence at Rome is consumption. In the early stages of this affection Sir James Clark has generally found the climate favorable. In bronchial affections, he says, the climate is very generally beneficial, especially in cases where there prevails great irritability of the bronchial membrane, with much sensibility to harsh cold winds. In chronic bronchitis, more especially when of the dry irritable kind, or complicated with irritation of the digestive organs, a residence at Rome produced the best effects; and in cases of this kind Sir James Clark considers it the most favorable residence on the continent. Chronic rheumatism is generally much relieved.

With persons disposed to apoplexy, or who have already suffered from paralytic affections, and valetudinarians of a nervous melancholic temperament, or subject to mental despondency, the climate of Rome, our author says, does not agree; nor is it proper for persons disposed to hemorrhagic diseases, or those who have suffered from intermittent fever.

*Naples.* In its general character the climate of Naples resembles that of Nice more than any other. Of this place as a residence for invalids, it is remarked that consumptive patients should certainly not be sent there. The qualities of its climate sufficiently mark it as a very unsuitable residence for this class of invalids; to the list of its defects must be added that of its topographical position which affords no proper places for exercise without such exposure as would prove highly injurious to delicate invalids. For chronic rheumatism, the climate is certainly inferior to that of Nice and Rome. Naples is however well suited, Sir James Clark remarks, as a winter residence for those who are labouring under general debility and deranged health without any marked local disease, and who require mental amusement and recreation rather than a mild equable climate.

*Malta.* No place in the south of Europe, Dr. Liddell thinks, can compete with Malta for a mild and bracing air in November, December, and part of January; and during the other winter and spring months, he thinks it is equal to any of them.

*Madeira.* When we take into consideration, says our author, the mildness of the winter and the coolness of the summer, together with the remarkable equality of the temperature during the day and night, as well as throughout the year, we may safely conclude that the climate of Madeira is the finest in the northern hemisphere. There is no place on the continent of Europe with which Sir James Clark is acquainted, where the pulmonary invalid could reside with so much advantage during the whole year as in Madeira. Although in his account of the climate of Madeira the author has confined himself to its influence on consumption, he thinks there can be no doubt of its being highly beneficial in several other diseases, more especially scrofula and bronchial affections. Were the accommodations for strangers at Teneriffe, and the means of communication between it and Madeira more frequent, many invalids, Sir James Clark thinks, might benefit greatly by passing the winter partly at Funchal and partly at Santa Cruz. Orotava is cooler but not so dry as Santa Cruz, and as a residence for invalids possesses many advantages over it.

*The Azores.* The Azores, situated in the centre of the northern Atlantic, nearly equidistant from the pole and the equator, and surrounded on all sides by a vast extent of ocean, present a climate purely oceanic, and afford one of the best examples of a mild, humid, equable climate to be met with in the northern hemisphere. It is slightly colder and more humid than Madeira, but probably even more equable. In diseases in which a soft soothing climate is indicated, that of the Azores will prove beneficial; thus in gastritic or inflammatory dyspepsia, and in bronchial irritation, accompanied with little secretion, it is indicated; on the other hand, in a relaxed state of the system, in those morbid conditions of the mucous membranes attended with copious discharges, and in an enfeebled state of the digestive organs, it will decidedly disagree.

Comparing Madeira, the Canaries, and the Azores, says our author, we find a gradual transition from the humid, soft, equable climate of the Azores, of which the mountains are covered to the very summit with evergreens, to that of arid, rocky Teneriffe, where the want of rain during the greater part of the year renders much of the island dry and sterile. Madeira presents an intermediate climate; less humid than the Azores, and less dry than Teneriffe during the winter, it has the advantage of a cooler summer than either.

*Bermuda.* The climate of Bermuda is variable and windy during the winter, and hot and oppressive in the summer. Compared with Madeira, which lies in the same parallel of latitude, the climate will be found much more unequal. The temperature of the two places during the winter may be much the same, but there is a wide difference in that of summer. The coolness of this season at Madeira forms a striking contrast with the oppressive heat of Bermuda. With so few advantages in point of climate, our author remarks, the Bermudas are not likely to become the resort of invalids from this country.

*The Bahama Islands* are not well calculated for the generality of invalids. The climate is not suited for consumptive patients on account of the rapid changes of temperature and the prevalence of winds often of a dry cold character. At the same time persons for whose cases a warm climate is indicated, Sir James Clark says, may pass the winter in the Bahamas safely; and residents in the West Indies might derive considerable benefit by a change to these islands for a few months during this season.

*West Indies.* The climate of the West Indies appears to be prejudicial in consumption, but it has been found to exert a favorable influence as a prophylactic. It is a remarkable fact, confirmed to Sir James Clark by Drs. Arnold and Musgrave, that persons obviously predisposed to consumption are rarely attacked by the indigenous fever. The cases of pulmonary consumption in which the climate of the West Indies promises advantage, says our author, are very few, and their character scarcely ascertained; while those in which it produces mischief are numerous and generally well marked. Of persons predisposed to the disease, a certain proportion are likely to be benefited by the climate; but the nature of the constitution should be well considered before it is recommended even as a prophylactic. The affections of the chest most likely to derive benefit from a residence in the West Indies are chronic diseases of the bronchial membrane in persons in whom the digestive organs are in a sound state. In stomach complaints the West Indies are very generally unfavorable. Neither has the climate been found favorable in chronic rheumatism. Calculous disease and scrofula are rare in the West Indies.

Jamaica from its size differs from the other islands in presenting more variety of climate. Of all the small West India islands, St. Kitt's as a high and Barbadoes as a low island appear to deserve the preference. But a more advantageous and better plan for the invalid than residing in any one island would be, according to our author, to cruise among or make short visits to the different islands. St. Kitt's from its situation among a group of islands is well calculated for the head-quarters of an invalid having such a plan in view.

In the Appendix, besides notices of the climate of the *Cape of Good Hope*, *Australia*, and *New Zealand*, there are some useful observations on the proper application of mineral waters as adjuvants to climate in the cure of some of the principal diseases treated of in the work. The whole is concluded with a more copious and elaborate set of comparative meteorological tables than are to be found anywhere else.

Everything in the work before us being so concisely yet so clearly stated, our jottings of the author's practical conclusions have been sufficient for the foregoing notice, the preparation of which has been to us at once an easy and an agreeable task. From the length of time the last edition has been out of print, and considering the interest which attaches to the subject, our readers will not consider as too much the space we have occupied.

In attestation of the soundness of the author's conclusions, no observation of ours is necessary. Their importance will be at cnce perceived by the reader. The work is one which, with great truth may be said, ought to be studied by every physician. From the perusal of it we have derived the greatest pleasure, and we can with equal truth add instruction. The principles inculcated are of the highest importance to mankind, and are advocated with all the ardour of one who pleads for truth. It is, to conclude, one of those works, too rare indeed, in which philosophy is attended by simplicity, and in which perfect elegance reigns throughout without the slightest affectation of ornament.

